# Blackburn Royal Infirmary

**Published:** 1924-05

**Authors:** Nathan A. Smith

**Affiliations:** General Supt. and Secretary


Manchester Royal Infirmary.

### Blackburn Royal Infirmary.

SIR,—I hasten to express our appreciation of the notice you have given to this Institution for the past year and wish to thank you sincerely for the prominence you have given to our report. With regard to your note of the total available beds being 152 and the average number occupied daily 156.12, I would point out that your inference is quite correct. The increased number has been caused by additional beds having been fixed in the wards during most of the year.

With regard to your last reference in the paragraph that the Report is without an index and that you had to read to the 18th page before you discovered the number of beds, I would point out that at page 3 of our Report you will find an index which we have designated "Contents."

NATHAN A. SMITH,
General Supt. and Secretary.

### Young People in Factories.

**The Home Secretary has appointed a committee to inquire into the working of the provisions of the Factory and Workshop Acts for the medical examination of young persons as to their fitness for employment in factories.**

